# Risk factors associated with Retinopathy of Prematurity development and progression

**DOI:** 10.1038/s41598-022-26229-4

**Published:** 2022-12-20

**Authors:** Nieves de las Rivas Ramírez, Guillermo Luque Aranda, Francisca Rius Díaz, Francisco Javier Pérez Frías, Tomás Sánchez Tamayo

**Affiliations:** 1 Department of Ophthalmology, Hospital de la Serranía, Ronda-San Pedro, Km 2, 29400 Ronda, Málaga Spain; 2 School of Medicine, Málaga University (Facultad de Medicina, Universidad de Málaga), Andalucía Tech, Campus de Teatinos s/n, 29071 Málaga, Spain; 3 Department of Ophthalmology, Hospital Regional Universitario de Málaga, 29010 Málaga, Spain; 4 Department of Preventive Medicine and Public Health, Biostatistics, School of Medicine, Málaga University, 29071 Málaga, Spain; 5 Department of Pediatrics, Hospital Regional Universitario de Málaga, 29010 Málaga, Spain; 6 Pediatrics Division, Málaga University, 29071 Málaga, Spain

**Keywords:** Risk factors, Retinopathy of prematurity, Epidemiology

## Abstract

Several studies propose that Retinopathy of Prematurity (ROP) is a multifactorial disorder implicating many prenatal and postnatal factors. The objective of our study was to determine the incidence and the risk factors that influenced ROP development and progression. We retrospectively compiled data of preterms with birth weight (BW) ≤ 1.500 g and/or gestational age (GA) < 32 weeks, or BW between 1.501 and 2.000 g and/or GA ≥ 32 weeks with oxygen supply > 72 h or unstable clinical course screened for ROP in Regional University Hospital of Málaga from 2015 to 2018. 202 infants (44.7%) developed ROP and 66 exhibited progression (32.7% of ROP infants). In the univariate analysis, many risk factors were associated with ROP. In the subsequent multivariate analysis, GA, oxygen therapy and weight at 28 days of life, mechanical ventilation duration, non-invasive ventilation, surfactant administration and late-onset sepsis were independently associated with the development. However, oxygen therapy duration, late-onset sepsis and weight at 28 days were associated with the progression. The ROP development and progression risk factors were different. Our results are important to facilitate screening, early diagnosis and ROP treatment while reducing unneeded examinations.

## Introduction

Retinopathy of prematurity (ROP), first described in 1942 by Terry^1^, is a vasoproliferative disorder of the premature infants retina and one of the major causes of childhood blindness worldwide^2^.

The retinal vasculature development starts at gestation (16 weeks) and completes at term^3^. ROP is a two-stage disorder that begins with the retinal vessels arrest in preterm babies exposed to high oxygen levels at birth^3^. The successive hypoxia brings about the angiogenic growth factors upregulation and the neovascularization risk^4^.

With the advancement in the preterm infants survival rate, the number of babies with higher ROP risk has increased, so early detection and treatment are becoming essential. Eye examinations are planned according to gestational age (GA), birth weight (BW) and ROP stage^5^.

Numerous studies suggest that ROP is a multifactorial disease involving a lot of prenatal and postnatal factors^6^ but the results are often controversial and few studies have discerned the ROP development risk factors from those of ROP progression.

The aim of this study was to determine the potential risk factors of ROP and establish their influence in ROP development and progression in order to find out the reason why in certain cases ROP progresses and in others it shows improvement.

## Methods

### Ethical approval and informed consent

The present study was approved by the Biomedical Research Ethics Committee of Andalusia and was carried out in accordance with the ethical principles for medical research outlined in the Declaration of Helsinki. Therefore, all medical data collected anonymously prior informed consent.

### Patient selection and study design

An observational retrospective study was conducted on 452 premature newborns who were examined between January 1, 2015 and December 31, 2018, at the level three Neonatal Intensive Care Unit (NICU) of the Regional University Hospital of Málaga (Spain).

Inclusion criteria were: BW ≤ 1.500 g and/or GA < 32 weeks, or BW between 1.501 and 2.000 g and/or GA ≥ 32 weeks with oxygen supply > 72 h or unstable clinical course as determined by the NICU neonatologist (apnea, neonatal acidosis, twin death, intraventricular hemorrhage, patent ductus arteriosus, sepsis, necrotizing enterocolitis or concurrent surgical interventions).

Participants were excluded from the study when they could not be examined until complete retinal vascularization, they presented severe systemic diseases, congenital anomalies or other ocular abnormalities that could affect ROP development or progression, died before eye examination or had incomplete medical data.

### Patient exam

All patients were examined under pharmacological mydriasis and topical anaesthesia by the same pediatric ophthalmology team using indirect ophthalmoscopy with indentation and a 20 diopter lens^5^. The RetCam Imaging System III (Clarity Medical Systems, Pleasanton, CA) was used to control the disease and treatment response^5^.

The infants were first examined at 4 to 6 weeks after birth. Subsequently, routine re-examination was performed every week depending on the retinal findings^5^. ROP was classified according to the International Classification of Retinopathy of Prematurity^7^, including the stage, zone and presence or absence of plus disease. ROP development was defined as the occurrence of any stage of ROP, and ROP progression was defined as prethreshold ROP or type-1 ROP (zone I, any stage ROP with plus disease, or zone I, stage 3 ROP without plus disease, or zone II, stage 2 or 3 ROP with plus disease), Threshold ROP (at least 5 contiguous clock-hours of extraretinal neovascularization or 8 cumulative clock-hours of extraretinal neovascularization in association with plus disease and location of the retinal vessels within zone I or II) or Aggressive Posterior ROP (AP-ROP) (vascularization ends in zone I or very posterior zone II and is accompanied by plus disease); treated conforming to Spanish ROP protocol^8^. The most advanced ROP stage recorded was assigned to each infant, without ROP differences between eyes. All patients were followed up until full retina vascularization^5^.

We considered 55 potential risk factors that may influence ROP and they were divided into birth, maternal, neonatal, hospitalization and ophthalmological factors. The full list of factors evaluated is given in Table 1.

**Table 1:** Potential risk factors for development and progression of ROP.

**Birth and maternal factors**		
In vitro fertilization (IVF)	Maternal diabetes mellitus (GDM)	Chorioamnionitis
Maternal age	Gestation (single or multiple)	Antenatal steroid therapy
Maternal hypertensive stages	Mode of delivery (vaginal, caesarean or instrumental)	Rupture of membranes (ROM) (h)
**Neonatal factors**		
Sex	Fetal heart rate alteration	Anemia
Race	Hypotension	Red blood cell transfusions
Gestational age (GA) (weeks)	Sepsis (early and late-onset)	Patent ductus arteriosus (PDA)
Birth weight (BW) (g)	Apnea	Surgical closure of PDA
APGAR score at 1 and 5 min	Pneumothorax	Moderate or severe bronchopulmonary dysplasia (BPD)
Cardiopulmonary resuscitation (CPR)	Respiratory distress syndrome (RDS)	Red blood cell transfusions
Intubation	Steroids for BDP	Patent ductus arteriosus (PDA)
**Hospitalization factors**		
Supplemental O_2_ therapy duration (days)	Non-invasive ventilation (NIV)	Intraventricular hemorrhage (IVH)
Mechanical ventilation (MV) duration (days)	Highest and minimum FiO_2_	Periventricular leukomalacia (PVL)
	Weight at 28 days (g)	Parenteral nutrition (PN) at 28 days
	Mechanical ventilation (MV)	Surfactant administration
Continuous positive airway pressure therapy (CPAP)	Supplemental O_2_ at 28 days	Necrotizing enterocolitis (NEC)
Nasal high-flow therapy	Total length of stay (days)	Spontaneous bowel perforation
High frequency oscillatory ventilation (HFOV)	Phototherapy	Cytomegalovirus (CMV) infection and treatment
**Ophthalmological factors**		
GA at ROP diagnosis (weeks)	Weight at ROP diagnosis (g)	Weight at ROP maximum stage (g)
	GA at ROP maximum stage (weeks)	

These variables were defined with regard to the criteria established by the Vermont Oxford working group^9^. Sepsis was defined as sepsis clinical signs and/or suggestive laboratory test results with a confirmatory blood culture, either early (in the first 72 h of life) or late (after the first 72 h of life).

### Statistical analysis

The enrolled neonates were divided into two subgroups: neonates who developed ROP or those who did not. Neonates who developed ROP were subsequently divided into: neonates who showed ROP progression or those who did not. To identify risk factors, each one was initially analysed by univariate analysis. The Pearson χ^2^ test and the T student test were used for categorical and numerical variables, respectively. Numerical variables were expressed as means ± standard deviation and categorical variables as absolute and relative frequencies.

Multivariate analysis using tree-building algorithms was then performed to identify the independent risk factors for ROP development and progression. Firstly, risk factors were divided into several groups (oxygen, ventilation, nutrition, anemia, comorbidities, resuscitation, ophthalmological factors and respiratory disease) and tree-building algorithm multivariate analysis was done. Secondly, we repeated the analysis including only the significative variables that we obtained in the previous analysis. Progression analysis was performed exclusively on patients who develop ROP.

All analyses were performed using the Statistical Package for Social Sciences (SPSS software version 25.0 (IBM Corp., Armonk, NY, USA). Statistically significance was defined as a *p* value < 0.05.

## Results

### Demographic characteristics

**Figure 1:**
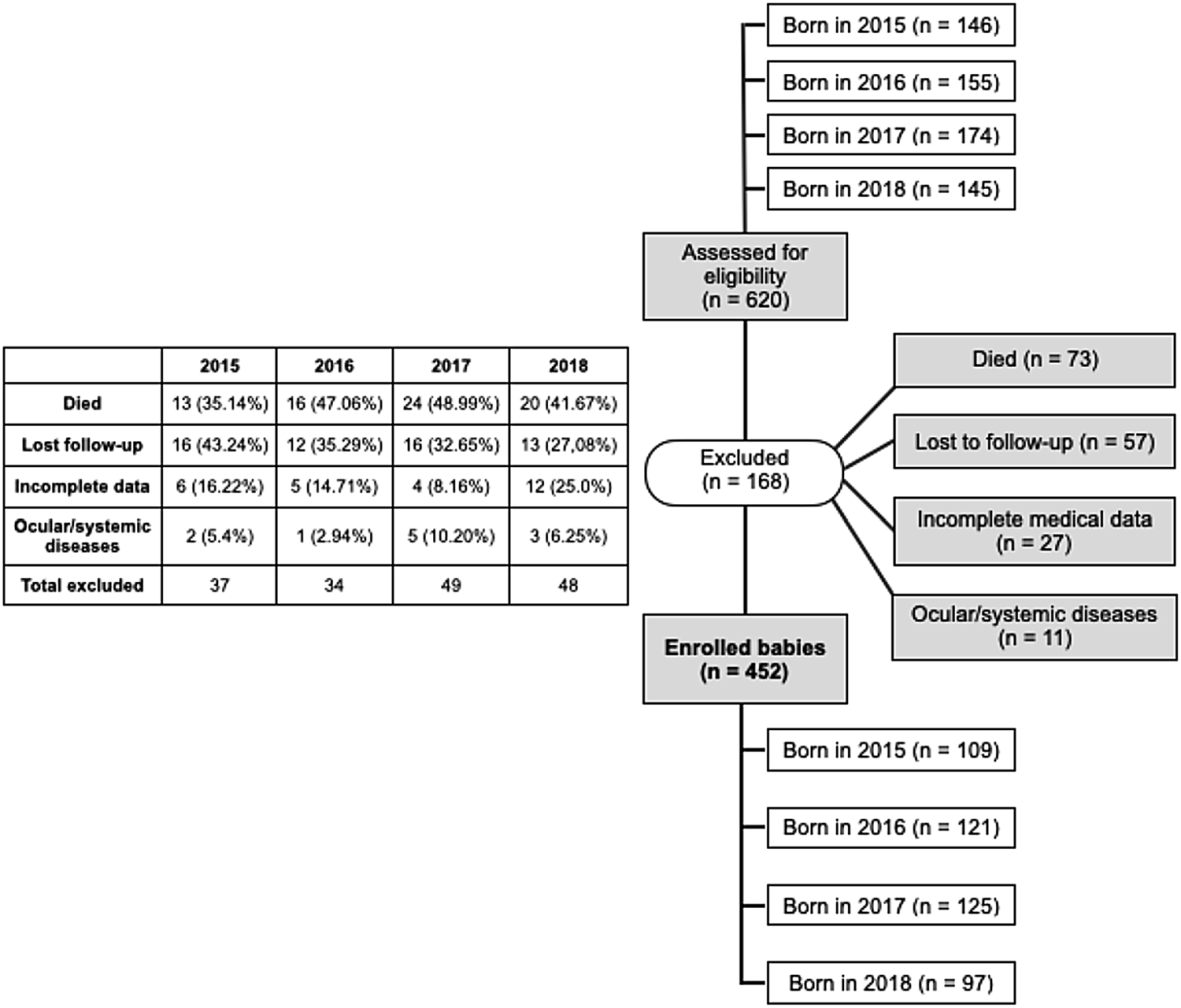
Flow diagram of the study. The chart on the left of the figure shows the exclusion reasons by year.

A total of 620 premature newborns were screened, of which 168 were excluded because they not meet the inclusion criteria (Fig. 1). The diagnoses of the 11 patients excluded due to ocular or systemic diseases were: 1 Down syndrome, 1 maternal human immunodeficiency virus infection, 1 congenital syphilis, 2 congenital cataracts that underwent surgery, 1 cystic fibrosis, 1 absent septum pellucidum, 1 fetal umbilical-porta-systemic venous shunt, 1 nephromegaly, 1 aorta coarctation. The mean GA at birth was 29.38 ± 2.53 weeks, the mean BW was 1187.19 ± 322.27 g, and the sex distribution was 228 (50.4%) males and 224 (49.6%) females.

### ROP characteristics

Of the 452 newborns ultimately included in the study, 202 (44.7%) developed ROP: 24 had zone I disease, 169 had zone II disease, and 9 had zone III disease; 45 had stage 1 (22.3%), 120 had stage 2 (59.4%), 33 had stage 3 (16.3%), 4 had stage 4 (2%). None of the neonates presented ROP at stage 5. AP-ROP was detected in 29 infants (14.4%).

Among the 202 infants that developed ROP, 66 progressed to ROP requiring treatment (14.6% of all of screened newborns and 32.7% of ROP developed newborns). There were 57 (12.6%) babies who underwent ROP treatment (14 intravitreal anti-VEGF, 33 laser photocoagulation therapy and 10 needed both treatments). The remaining 9 patients didn’t need treatment due to spontaneous improvement of retinopathy in ophthalmological exam. Table 2 shows the rates of ROP development and progression, AP-ROP and treatment of the time lapse under research.

**Table 2:** ROP development and progression, AP-ROP and treatment from 2015 to 2018.

	Born 2015	Born 2016	Born 2017	Born 2018	*p*
ROP development	35 (32.1%)	66 (54.5%)	56 (44.8%)	45 (46.4%)	0.008*
ROP progression	8 (7.3%)	26 (21.5%)	21 (16.8%)	11 (11.3%)	0.015*
Presence AP-ROP	0 (0.0%)	10 (8.3%)	12 (9.6%)	7 (7.2%)	0.016*
ROP treatment	5 (4.6%)	22 (18.2%)	20 (16.0%)	10 (10.3%)	0.009*

*p < 0.005.

### Univariate analysis for ROP development and progression

After univariate analysis, various factors were identified as significant risk factors for ROP development and progression (Tables 3, 4 and 5). There were no differences in sex, race, fetal heart rate, early sepsis, phototherapy, IVF, maternal age, maternal hypertensive stages, GDM, gestation, mode of delivery, antenatal steroid therapy and PVL for ROP development and progression. In addition to, no differences were found in GA and weight at ROP maximum stage for ROP progression.

**Table 3:** Significant ophthalmological, birth and maternal risk factors after univariate analysis for ROP development and progression.

Ophthalmological, birth and maternal factors	Development			Progression		
	No	Yes	*p*	No	Yes	*p*
GA at ROP diagnosis	–	–	–	35.19 ± 2.34	34.26 ± 1.89	0.005*
Weight at ROP diagnosis (g)	–	–	–	1921.49 ± 541.79	1663.14 ± 352.60	0.001*
ROM (h)	59.74 ± 227.82	46.24 ± 126.55	0.452	31.09 ± 94.61	77.45 ± 171.65	0.044*
Chorioamnionitis	49 (19.6%)	58 (28.7%)	0.023*	35 (25.7%)	23 (34.8%)	0.179

*GA* gestational age (weeks), *ROM* rupture of membranes (h). *p < 0.005.

**Table 4:** Significant neonatal risk factors after univariate analysis for ROP development and progression.

Neonatal factors	Development			Progression		
	No	Yes	*p*	No	Yes	*p*
GA (weeks)	30.74 ± 1.77	27.69 ± 2.32	0.0001*	28.32 ± 2.23	26.39 ± 1.95	0.0001*
*BW (g)*	1350.20 ± 248.91	985.45 ± 286.91	0.0001*	1054.11 ± 275.22	843.95 ± 258.93	0.0001*
APGAR score 1’	6.82 ± 2.04	5.85 ± 2.20	0.0001*	6.20 ± 2.13	5.12 ± 2.17	0.001*
APGAR score 5’	8.62 ± 1.35	7.99 ± 1.62	0.0001*	8.22 ± 1.56	7.50 ± 1.64	0.003*
CPR	121 (48.4%)	142 (70.3%)	0.0001*	88 (64.7%)	54 (81.8%)	0.013*
Intubation	31 (12.4%)	102 (50.5%)	0.0001*	54 (39.7%)	48 (72.7%)	0.0001*
Hypotension	7 (2.8%)	53 (26.2%)	0.0001*	25 (18.4%)	28 (42.4%)	0.0001*
Moderate/severe BDP	11 (4.4%)	64 (31.7%)	0.0001*	26 (40.6%)	38 (59.4%)	0.0001*
Steroids for BDP	7 (2.8%)	61 (30.2%)	0.0001*	33 (24.3%)	28 (42.4%)	0.0001*
RDS	50 (20.0%)	72 (35.6%)	0.0001*	43 (31.6%)	29 (43.9%)	0.086
Late-onset sepsis	57 (22.8%)	100 (49.5%)	0.0001*	54 (39.7%)	46 (69.7%)	0.0001*
Apnea	36 (14.4%)	84 (41.6%)	0.0001*	49 (36.0%)	35 (53.0%)	0.021*
Pneumothorax	3 (1.2%)	7 (3.5%)	0.104	2 (1.5%)	5 (7.6%)	0.026*
Anemia	45 (18.0%)	127 (62.9%)	0.0001*	74 (54.4%)	53 (80.3%)	0.0001*
Red blood cell transfusions	25 (10.0%)	79 (39.1%)	0.0001*	44 (32.4%)	35 (53.0%)	0.005*
PDA	33 (13.2%)	91 (45.0%)	0.0001*	49 (36.0%)	42 (63.6%)	0.0001*
Surgical closure PDA	3 (1.2%)	24 (11.9%)	0.0001*	9 (6.6%)	15 (11.9%)	0.001*

*GA* gestational age (weeks), *BW* birth weight (g), *CPR* cardiopulmonary resuscitation, *DBP* bronchopulmonary dysplasia, RDS respiratory distress syndrome, *PDA* patent ductus arteriosus. *p < 0.005. Percentages refer to children with the risk factor.

**Table 5:** Significant hospitalization risk factors after univariate analysis for ROP development and progression.

Hospitalization factors	Development			Progression		
	No	Yes	*p*	No	Yes	*p*
O_2_ duration (days)	9.36 ± 13.86	42.34 ± 38.47	0.0001*	29.76 ± 30.43	68.26 ± 40.51	0.0001*
Minimum FiO_2_	21.30 ± 1.19	22.58 ± 4.68	0.0001*	22.29 ± 4.22	23.18 ± 5.50	0.250
Highest FiO_2_	31.08 ± 14.51	38.33 ± 20.70	0.0001*	36.05 ± 19.25	43.15 ± 22.85	0.035*
MV duration (days)	6.12 ± 5.65	18.67 ± 17.35	0.0001*	14.51 ± 13.19	24.64 ± 20.70	0.002*
Length of stay	39.34 ± 15.21	74.40 ± 30.14	0.0001*	64.96 ± 25.86	93.86 ± 29.13	0.0001*
Weight 28-days	1848.78 ± 315.78	1356.52 ± 394.66	0.0001*	1452.29 ± 369.53	1159.18 ± 373.24	0.0001*
CPAP	212 (84.8%)	192 (95.0%)	0.0001*	127 (93.4%)	65 (98.5%)	0.117
MV	51 (20.4%)	125 (61.9%)	0.0001*	72 (52.9%)	53 (80.3%)	0.001*
O_2_ 28 days	23 (9.2%)	115 (56.9%)	0.0001*	62 (45.6%)	53 (80.3%)	0.0001*
Nasal high-flow therapy	54 (21.6%)	97 (48.0%)	0.0001*	54 (39.7%)	43 (65.2%)	0.001*
HFOV	22 (8.8%)	68 (33.7%)	0.0001*	29 (21.3%)	39 (59.1%)	0.0001*
NIV	15 (6.0%)	82 (40.6%)	0.0001*	43 (31.6%)	39 (59.1%)	0.0001*
Surfactant administration	60 (24.0%)	126 (62.4%)	0.0001*	74 (54.4%)	52 (78.8%)	0.001*
IVH	30 (12.0%)	64 (31.7%)	0.0001*	35 (25.7%)	29 (43.9%)	0.009*
PN 28 days	88 (35.2%)	78 (38.6%)	0.454	45 (33.1%)	33 (50.0%)	0.021*
NEC	2 (0.8%)	16 (7.9%)	0.0001*	6 (4.4%)	10 (15.2%)	0.008*
Spontaneous bowel perforation	1 (0.4%)	11 (5.4%)	0.001*	5 (3.7%)	6 (9.1%)	0.112
Positive CMV infection	3 (1.2%)	15 (7.4%)	0.001*	7 (5.2%)	8 (12.1%)	0.076
CMV treatment	0 (0.0%)	11 (5.4%)	0.0001*	5 (3.7%)	6 (9.1%)	0.112

*MV* mechanical ventilation, *CPAP* continuous positive airway pressure therapy, *HFOV* High frequency oscillatory ventilation, *NIV* non-invasive ventilation, *NEC* necrotizing enterocolitis, *CMV* cytomegalovirus. *p < 0.005. Percentages refer to children with the risk factor.

### Multivariate analysis for ROP development and progression

**Figure 2:**
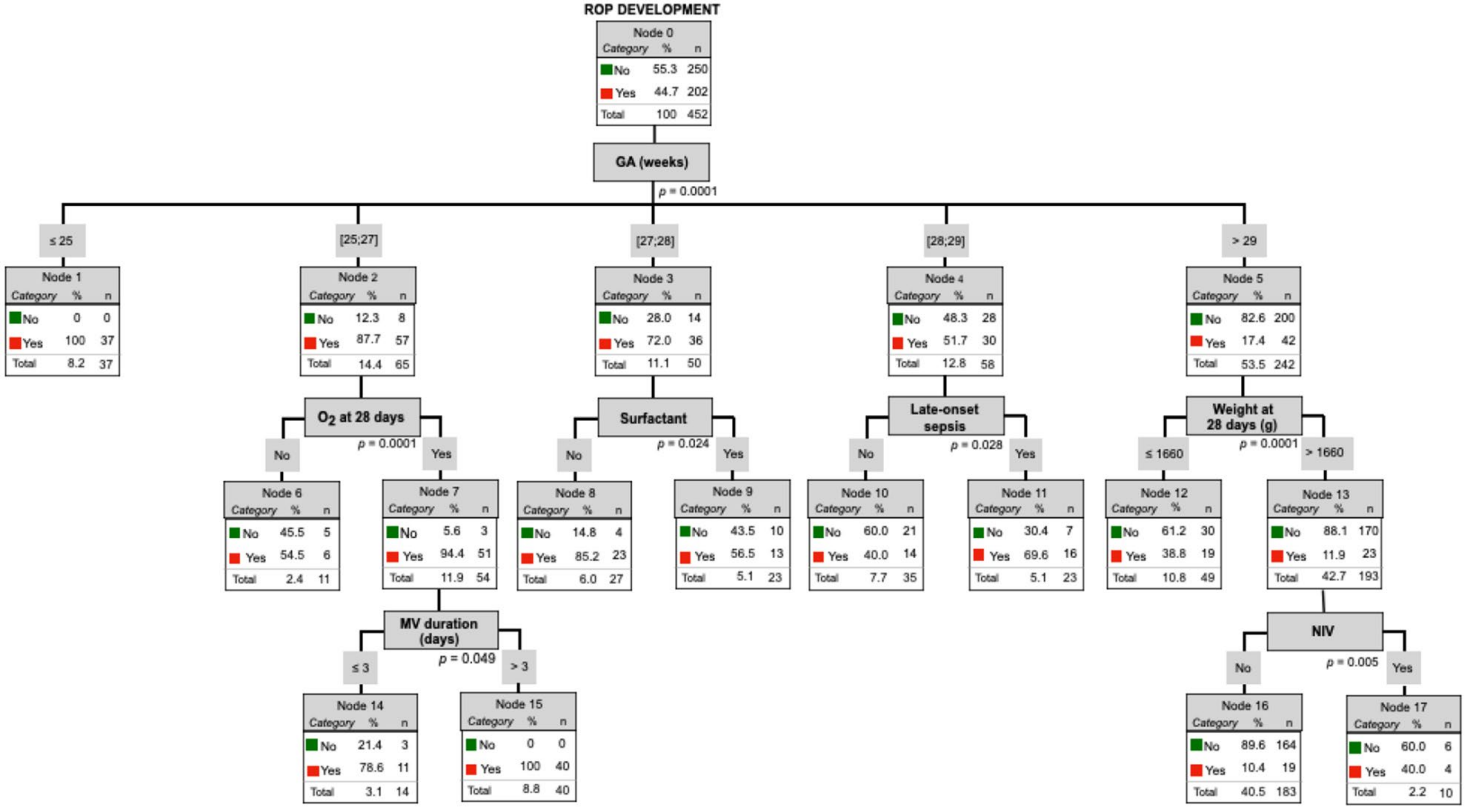
Tree algorithm for ROP development. *GA* gestational Age (weeks), *MV* mechanical Ventilation, *NIV* non-invasive ventilation. *p < 0.005.

**Figure 3:**
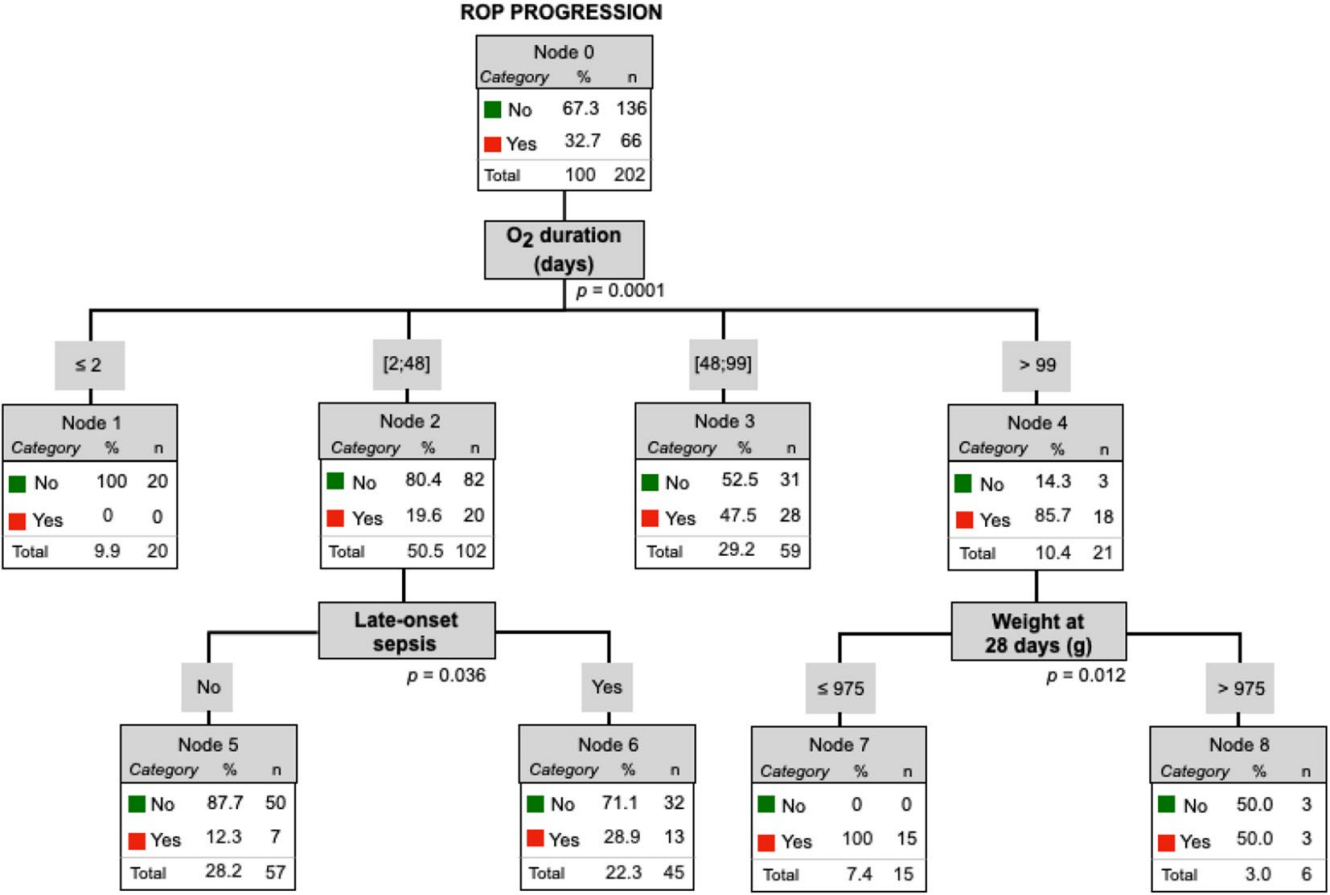
Tree algorithm for ROP progression. *p < 0.005.

All the significant variables obtained in the different groups trees exposed previously were included in the following tree algorithm for ROP development (Fig. 2) (GA, oxygen at 28 days of life, NIV, MV duration, BW, weight at 28 days of life, late-onset sepsis, CMV, treatment for CMV, NEC, hypotension, intubation, steroids for BDP, apneas, surfactant administration, IVH and anemia) and progression (Fig. 3) (duration of oxygen therapy, nasal high-flow therapy, HFOV, weight at 28 days of life, late-onset sepsis, ROM, weight at ROP diagnosis, hypotension, PDA, intubation, steroids for BDP, surfactant administration, IVH and anemia).

Final multivariate analysis with the significative risk factors of each group for ROP development (Fig. 2) evidenced that the GA have the strongest influence on the development of ROP; increasing the risk of ROP development as the GA decreases. In those patients with GA between 25–27 weeks the need of supplemental oxygen therapy increases the ROP development risk to 94.4%, and it reaches 100% in those babies who need more than 3 days of MV. In preterms with 27–28 weeks of GA, surfactant administration decreases the risk of ROP development until 56.5% and in those with 28–29 weeks of GA the late-onset sepsis increases ROP risk (69.6%). However, in GA > 29 weeks group, the weight at 28 days of life is the risk factor that firstly influence on the development, showing lower risk those with more than 1660 g, with an increased risk of ROP if they need NIV.

Final multivariate analysis with the significative risk factors of each group for ROP progression (Fig. 3) demonstrated that the supplemental oxygen therapy duration has the strongest influence on the progression of ROP; increasing the ROP progression risk as the number of days of oxygen increases. In preterms who need oxygen between 2–48 days, the late-onset sepsis diagnosis increases the risk of ROP progression to 28.9%. In preterms who need oxygen more than 99 days with ≤ 975 g of weight at 28 days of life ensures ROP progression.

## Discussion

The ROP incidence differs between countries with screening criteria guidelines ranging from < 30 to 37 weeks of GA and from 1.000 to 2.500 g of BW^10^. Our study showed ROP development in 44.7% of screened newborns and ROP progression in 32.7% of ROP newborns, as well as differences in the risk factors that influenced ROP development and progression.

Low GA and BW are the two strongest known risk factors for ROP development^11^. In our study, the mean GA was 27.69 ± 2.32 weeks among infants with ROP development and 26.39 ± 1.95 weeks among infants with ROP progression, significantly lower that among infants without ROP development or progression. Also, GA and weight at ROP diagnosis were significantly lower among infants with ROP progression; instead, we didn’t find significantly association between GA and weight at ROP maximum stage and risk of ROP.

Similar to other studies, the mean weight at 28 days of life was significantly lower among those who showed ROP development or progression in this study. Although Chaves-Samaniego et al.^12^ reported that the risk of developing ROP requiring treatment decreased with increasing weight gain ≥ 14 g/day in the first 4 weeks of life, they recommend to take into account GA, BW, time of MV and comorbidity to evaluate ROP risk. Other neonatal demographic risk factors such as sex and race were not significantly associated with risk of ROP.

Omega-3 long-chain polyunsaturated fatty acids have been shown to reduce pathological angiogenesis in a ROP animal model^13^. Several studies have associated fish oil lipid emulsion for parenteral nutrition with lower incidence of any stage of ROP^13–15^. We found that infants with PN showed lower rate of ROP progression. However, prolonged PN has been suggested as a ROP risk factor^16,17^.

Several studies have reported that lower Apgar scores, as a general indicator of poor neonatal health, may be associated with higher rates of ROP^11^. However, on multivariate regression analysis, the relationship was not significant in most cases, as occurs in our work. Further, neonatal CPR was associated with increased ROP development and progression risk. It is likely that, in addition to the underlying systemic disease that made resuscitation necessary, the worsening patient general condition or the possibility of hypoxia periods followed by hyperoxia after CPR are related to the anormal retinal vasculature development in these patients. Similarly, the presence of arterial hypotension during first week of life was related to a higher frequency of ROP development and progression, it could be possibly associated with patient worsening general condition as well as hypoperfusion and subsequent reperfusion problems.

Many studies have indicated that sepsis was closely related to the appearance of any stage of retinopathy and associated with AP-ROP^18,19^. In our work, there were no differences in early-onset sepsis. However, late-onset sepsis was risk factor for ROP development and progression. Systemic inflammation may impare angiogenesis as well as hypotension and oxygen saturation fluctuation might affect the retinal perfusion and lead to retinal ischemia. The pathophysiology of NEC may implicate innate immune responses to intestinal microbiota, inducing inflammation that affects retinal angiogenesis therefore many studies have shown that it may increase ROP risk^20,21^, as in our work.

The mechanism that describes CMV influence on retinal vasculature remains unknown but recent studies show how the virus can activate the cytokines paracrine pathway that influences endothelial cells function and stimulates the liberation of pro-angiogenic factors, stimulating retinal inflammation and angiogenesis and leading to ROP development or progression^22^. In our study, there was an association with ROP development, being ROP more frequent in infection confirmed cases and in those requiring treatment.

It has been suggested that neonatal respiratory diseases are closely related to the development of ROP. Infants with apnea are more likely to require MV and supplementary oxygen, and may be more likely to develop ROP. Also, BPD and RDS have been associated with ROP. RDS is caused by surfactant deficiency so newborns with RDS may require MV and oxygen therapy. Not surprisingly, RDS is associated with increased risk of ROP. In this work all of them were risk factors for development and progression of the disease and pneumothorax was found to be associated with ROP progression. Many infants with RDS require surfactant administration. Some studies have suggested that surfactant therapy is closely related to ROP^23,24^, which can be interpreted as higher ROP risk in infants with RDS requiring treatment. In our work, we stated that surfactant administration is associated with higher risk of ROP development and lesser risk of progression.

The mean duration of oxygen therapy was significantly higher among infants with ROP development and progression as well as minimum and highest FiO_2_ levels. Regarding assisted ventilation, we found that any modality (HFOV, MV or NIV) and its duration were related to the development and progression of ROP. Similarly, the need of ventilatory support and/or oxygen at 36 weeks, which defines moderate/severe BDP, was also associated with an increased risk of ROP development and progression. These cases are patients with chronic respiratory disease in whom episodes of desaturation and subsequent hyperoxia are frequent during oxygen treatment, this could explain the increased risk.

Longer length of stay has been associated with ROP^17^. This may be due to length of stay is an indicative for cumulative illness. Our work revealed higher risk of ROP development and progression.

In addition, anemia and blood transfusions have also been implicated as ROP risk factors. A possible explanation could be that adult-type hemoglobin has a lower oxygen affinity and may cause excess oxygen release in the retinal tissue^25^. Other explanation could be that the iron load from transfusions may catalyze the formation of reactive oxygen species and accelerate oxidative damage, leading to ROP^25^. In our work, anemia and blood transfusions were associated with ROP development and progression but were no longer associated after multivariate analysis, as occurs in most of the recent studies^26^.

In infants with PDA, reduced perfusion due to systemic blood flow by-pass might result in retina hypoxia and might predispose to ROP development or progression**.** It has been reported associations between PDA and neonatal diseases such as NEC, IVH and BDP^27,28^ Similar to other studies^29^, PDA was an independent risk factor of ROP in our work. Furthermore, management of PDA may also be associated with ROP. Beharry et al.^30^ found that indomethacin, used as PDA treatment, could influence retinal neovascularization in animal models.

Regarding IVH, we observed a higher risk of ROP development without higher progression risk associated. IVH and ROP are both associated with immature vasculature and unstable oxygen supply^31^. In this context, iron molecules are released, which especially in the presence of oxygen excess, produce free radicals liberation.

Various studies have suggested that the pathophysiologic events that predispose preterm neonates to ROP may begin before delivery^6^. Ahn et al.^32^ reported the association of chorioamnionitis with ROP and AP-ROP. A meta-analysis of 27 studies revealed that chorioamnionitis was significantly associated with ROP by univariate analyses, but no association was found on multivariate analysis correcting for GA, as in our work^33^. Woo et al.^34^ investigated the relationship between cytokine levels in cord blood and perinatal factors and ROP in GA-matched preterm babies showing that cord blood cytokine levels are not associated with the risk of ROP, whereas increased maternal blood leukocyte count and low Apgar score are associated with an elevated risk of ROP. They also found that increased levels of inflammatory and angiogenic mediators in the amniotic fluid are independently associated with the development and progression of ROP^35^.

From one standpoint, multivariate analysis for ROP development showed that the GA has the strongest influence on the development of ROP; increasing the risk of ROP development as the GA decreases. That risk was subsequently modified depending on the GA group and the presence or not of other variables such as oxygen and weight at 28 days of life, MV duration, NIV, surfactant administration and late-onset sepsis. Otherwise, multivariate analysis for ROP progression demonstrated that the duration of supplemental oxygen therapy has the strongest influence on the progression of ROP; increasing the risk as the number of oxygen days increases and being modified depending on the association or not of other variables such as late-onset sepsis and weight at 28 days of life.

The main strength of our study is the large representative sample, which gives more faithful results. However, our results should be interpreted taking into consideration the following limitations: First, all the known risk factors could not be added to this study. Second, it was conducted in a single NICU, although it is a level three unit; nonetheless, this study provides relevant data on the ROP incidence and risk factors of the Spanish population and it may be used as a basis for future multicenter trials. Additional investigation is necessary to identify the risk factors role and validate them in other populations ( Supplementary Information).

## Conclusions

The reason why ROP development factors differs from ROP progression factors remains unclear. GA, MV duration, supplemental oxygen therapy at 28 days of life, NIV, surfactant administration, late-onset sepsis and weight at 28 days of life influence ROP development, whereas duration of supplemental oxygen therapy, late-onset sepsis and weight at 28 days of life are risk factors for ROP progression.

Our study suggests that, on one hand, GA is the first risk factor to determine ROP development risk; on the other hand, the duration of supplemental oxygen therapy is the first predictor of ROP worsening. These findings may help pediatricians and ophthalmologists to evaluate the risk of ROP development and progression and determine the appropriate timing of examinations and treatment.

## Supplementary Information

Supplementary Information.

## Data Availability

The datasets generated during and/or analysed during the current study are available from the corresponding author on reasonable request.
